# Low Dose Cranial Irradiation-Induced Cerebrovascular Damage Is Reversible in Mice

**DOI:** 10.1371/journal.pone.0112397

**Published:** 2014-11-13

**Authors:** Nikolett Sándor, Fruzsina R. Walter, Alexandra Bocsik, Petra Sántha, Boglárka Schilling-Tóth, Violetta Léner, Zoltán Varga, Zsuzsanna Kahán, Mária A. Deli, Géza Sáfrány, Hargita Hegyesi

**Affiliations:** 1 Division of Molecular Radiobiology and Biodosimetry, “Frédéric Joliot-Curie” National Research Institute for Radiobiology and Radiohygiene, Budapest, Hungary; 2 Biological Barriers Research Group, Institute of Biophysics, Biological Research Centre, Hungarian Academy of Sciences, Szeged, Hungary; 3 Department of Oncotherapy, University of Szeged, Szeged, Hungary; 4 Department of Morphology and Physiology, Faculty of Health Care, Semmelweis University, Budapest, Hungary; 5 Doctoral Schools of Pathological Sciences, Semmelweis University, Budapest, Hungary; National Cancer Institute, United States of America

## Abstract

**Background:**

High-dose radiation-induced blood-brain barrier breakdown contributes to acute radiation toxicity syndrome and delayed brain injury, but there are few data on the effects of low dose cranial irradiation. Our goal was to measure blood-brain barrier changes after low (0.1 Gy), moderate (2 Gy) and high (10 Gy) dose irradiation under *in vivo* and *in vitro* conditions.

**Methodology:**

Cranial irradiation was performed on 10-day-old and 10-week-old mice. Blood-brain barrier permeability for Evans blue, body weight and number of peripheral mononuclear and circulating endothelial progenitor cells were evaluated 1, 4 and 26 weeks postirradiation. Barrier properties of primary mouse brain endothelial cells co-cultured with glial cells were determined by measurement of resistance and permeability for marker molecules and staining for interendothelial junctions. Endothelial senescence was determined by senescence associated β-galactosidase staining.

**Principle Findings:**

Extravasation of Evans blue increased in cerebrum and cerebellum in adult mice 1 week and in infant mice 4 weeks postirradiation at all treatment doses. Head irradiation with 10 Gy decreased body weight. The number of circulating endothelial progenitor cells in blood was decreased 1 day after irradiation with 0.1 and 2 Gy. Increase in the permeability of cultured brain endothelial monolayers for fluorescein and albumin was time- and radiation dose dependent and accompanied by changes in junctional immunostaining for claudin-5, ZO-1 and β-catenin. The number of cultured brain endothelial and glial cells decreased from third day of postirradiation and senescence in endothelial cells increased at 2 and 10 Gy.

**Conclusion:**

Not only high but low and moderate doses of cranial irradiation increase permeability of cerebral vessels in mice, but this effect is reversible by 6 months. *In-vitro* experiments suggest that irradiation changes junctional morphology, decreases cell number and causes senescence in brain endothelial cells.

## Introduction

Radiation therapy is a well-known risk factor for injury of central nervous system (CNS) [1]. Neurocognitive functions are altered in cancer patients, especially in children treated with prophylactic cranial radiation in acute lymphoid leukemia [2], [3]. The CNS is still immature and in a state of rapid development in children, therefore more sensitive to X-ray radiation doses as low as 1–2 Gy or less [4]. Recently, the late side effects of low or moderate doses of ionizing radiation (range of 0.01–2 Gy) on the CNS have been under consideration, and the risk of computer tomography (CT) radiation is reevaluated [5]. There are few studies investigating the effects of low dose X-ray radiation on the CNS of young animals which describe temporary changes [6], [7].

Whole-brain irradiation in humans and animals lead to late structural changes, such as vascular damage, demyelination and white matter necrosis, as well as functional alterations including cognitive impairment [8], [9]. Apoptosis of endothelial, neural and glial cells was suggested as the primary mechanism of late effects of whole brain high dose radiation treatment [10]. Oxidative stress and neuroinflammation mediate radiation-induced secondary cell damage that leads further endothelial dysfunction, disruption of the blood-brain barrier (BBB), inhibition of cell regeneration, demyelination and tissue necrosis [10]. The BBB due to its unique structure limits the transport of a variety of harmful molecules from the blood to brain parenchyma and protects the CNS, while BBB dysfunction contributes to CNS pathologies [11], [12]. The molecular and cellular events that trigger radiation-induced endothelial damage [13] are still unclear, but can be potential therapeutic targets to treat BBB injury related to clinical ionizing radiation [14] and prevent secondary neuronal damage.

In the present study, the effect of irradiation on BBB damage and repair were examined in murine *in vivo* and *in vitro* model systems. We addressed the hypothesis that low dose local head irradiation caused cerebrovascular damage, decreased the ability of endothelial repair from circulating progenitors, and cell aging may be one of the mechanisms of the radiation-induced endothelial injury. Bone marrow derived cells contribute to repair and neovascularization following cranial radiation [15], [16] and circulating endothelial progenitors (CEPs) are successfully used for the therapy of cardiovascular and peripheral vascular diseases [17]. CEPs have the potential to promote postnatal vasculogenesis in adults and vascular regeneration after irradiation [16]. Therefore stress response and damage induced by radiation in stem/progenitor cells may be linked to attenuated endothelial regeneration and vascular disease. Bone marrow derived CEPs were identified as cellular markers of endothelial repair in the blood [18]. Ionizing radiation sensitize diverse types of stem cells and induces cardiovascular diseases, therefore we hypothesized that radiation-induced cerebrovascular dysfunction is associated with changes in the number of CEPs contributing to impaired vascular homeostasis. The effect of low dose cranial radiation on levels of CEPs were not studied yet, therefore in our work CEP abundance in blood was examined in parallel with BBB permeability after single cranial irradiation in mice.

To reveal the mechanism of post-irradiation BBB injury, the effect of ionizing radiation was studied in cells. Brain endothelial cells are major targets of X-ray exposure and participate in irradiation-induced BBB disruption, similarly to postirradiation oligodendroglia injury and subsequent demyelinization *in vivo* [13], [18]–[21]. There are only a few studies detailing the mechanism of radiation on cultured cells of the CNS. X-ray exposure causes activation of microglia and cell death in neurons [22]. Irradiation of astroglia cultures alone results in cell cycle arrest and reduced proliferation [23], while irradiation of astroglia-microglia mixed cultures induces astrogliosis [22], [24], [25]. There is only one study which examines the effects of moderate and high dose X-ray radiation on a culture model of the BBB [26]. High doses of radiation induce permeability elevation, junctional morphology changes, cell density decrease and formation of actin stress fibers in bovine cerebral endothelial cells [26]. Radiation also induces endothelial senescence, but its relation to BBB disruption is not established. In cellular senescence endothelial cells lose their ability to proliferate while they can stay metabolically active [27].

Moderate and chronic low dose of gamma irradiation induces senescence in rat cerebral endothelial cells and human umbilical vein endothelial cells along with impaired proliferative capacity [28]–[30]. Effects of single low dose of irradiation corresponding to CT or diagnostic X-ray doses were not yet studied on a relevant BBB culture model in detail, therefore irradiation-induced changes in brain endothelial function and morphology were examined in an *in vitro* mouse primary co-culture model of the BBB established and characterized in our laboratory [31]–[33].

The main aim of the study was to reveal changes in BBB permeability, brain endothelial function and morphology, and endothelial repair induced by low (0.1 Gy) dose of X-ray irradiation as compared to moderate (2 Gy) and high (10 Gy) doses using *in vivo* and *in vitro* experiments. To reveal age dependence, both adult (10 weeks old) and infant (10 days old) mice were examined. Reversibility of the effects was studied by following BBB changes from 1 day to 6 months. The *in vitro* co-culture BBB model allowed us to study the direct effect of low, moderate and high dose irradiation on barrier integrity, junctional morphology, cell number and senescence of mouse brain endothelial cells.

## Materials and Methods

### Ethics Statement

All animal studies were done according to the 1998. XXVIII. Hungarian law about animal protection and welfare. Formal approvals for animal studies have been obtained from the local Hungarian animal health authorities (Permit numbers: 22.1/2703/3/2011 and XVI./834/2012).

### Materials

All reagents were purchased from Sigma-Aldrich Ltd., Hungary, unless otherwise indicated.

### Animals

Female and male C57BL/6 mice were purchased from Janvier labs (Le Genest-Saint-Isle, France) and bred at our institute. Infant (10-day-old) and adult (10-week-old) mice were used for the experiments. The developmental stage of 10-day-old mice corresponds to that of human infants. The body growth in mice is completed at about postnatal day 50, therefore animals at postnatal day 70 are considered adult. For the experiments both female and male mice in equal number were used in all treatment and age groups. Animals were housed in individual cages (maximum 6 animals/cage) and were provided with standard food and water *ad libitum*. Infant mice were kept with their mothers until weaning.

### Local Cranial Irradiation

Mice were irradiated locally on their heads with single doses of 0.1, 2 and 10 Gy of X-rays as indicated at particular experiments. Cranial irradiation was performed using THX-250 X-ray machine that generated 250 kV X-rays with 1 mm Cu at a dose rate of 1.03 Gy/min. Mice were anesthetized with intraperitoneal injection of ketamine/xylazine (80 and 10 mg/kg bodyweight, respectively), and were placed in a prone position. A round lead lid of 1.1 cm thickness was used for irradiation, which had triangular holes for the head of the animals. Separate lids were used for the young and adult animals, where the dimensions of the holes were adapted to the head of the mice. The body of anesthetized animals was wrapped in a plastic bag and the bag was fixed on the inner surface of the lid. The animal was positioned in a way that only the head was in the irradiation field. The eyes, mouth and neck were under the lead cover; only the central part of the head was irradiated. Sham-irradiated animals (0 Gy group) were subjected to the same procedure as the irradiated ones.

### Irradiation of Cell Cultures

Primary mouse brain endothelial cells (MBECs) and primary mouse mixed glia cultures were irradiated using a linear accelerator (Siemens Primus; Siemens Medical Solutions, USA). Glial cells were irradiated in the co-culture model prepared for the permeability tests and kept in co-culture until the GFAP immunostaining was performed. The cultures were irradiated with 6 MV energy photon beams with opposing field technique. To achieve homogeneous dose distribution in the sample 2 cm thick PMMA sheets were applied above and under the plates. The dose rate at the center of the plates was 3.15 Gy/min for each beam. Doses (0.1, 2 and 10 Gy) were the same as applied during experiments *in vivo*. Changes in barrier integrity, cell number and cell morphology were investigated 1, 2, 3 and 5 days after radiation treatment. Shamirradiated (0 Gy) cells under the same conditions served as control in all experiments.

### Assessment of Blood-Brain Barrier Disruption In Vivo

Permeability of the BBB was quantified by the extravasation of Evans blue, a marker of albumin penetration [34], [35]. Evans blue dye (4% in saline) was administered *ip*. (4 ml/kg). This dye bounds tightly to albumin in blood and tissue fluids when injected to the body or when mixed *in vitro* with albumin solution and can be used as a quantitative marker for this protein [36]. Mice in deep anesthesia received a brief cardiac perfusion with isotonic saline 1 h post-injection. The cerebrum and cerebellum were removed and weighed. Formamide (0.5 ml) was added to the brain samples which were incubated overnight at 65°C. Supernatants were obtained by centrifugation (10,000×*g*, 10 min) and Evans blue content was measured with fluorescent plate reader at 620/680 nm wavelengths (Synergy HT, Biotek, USA). The amount of Evans blue present in the tissue samples was quantified using a linear regression standard curve derived from seven concentrations of the dye and expressed as ng/mg tissue.

### Measurement of Body Weight

Mice were housed under identical conditions to observe their feeding and drinking after treatment. Body weights were recorded at 1, 4 and 26 weeks postirradiation.

### Endothelial Progenitor Assay

Peripheral blood mononuclear fraction (PBMCs) was isolated by Histopaque-1084 density gradient centrifugation and cultured on square glass coverslips (22 mm×22 mm) coated with 0.2% gelatin. Circulating endothelial colony forming progenitor cells (CEPs) are derived from adherent PBMCs [37] when cultured for 14 to 21 days in endothelial conditions (EBM2 growth medium; EGM2-BulletKit, Lonza, Switzerland). CEP colonies were identified as multiple thin, flat cells growing out from a central cluster of rounded cells. CEP colonies at the end of the 2-weeks culture period consisted of 50 or more cells. Colonies were counted manually in a minimum of 2 coverslips/mice by independent blinded observers and the results were expressed as CEP-CFU/PBMCs ×10^5^. Blood-derived CEPs were fixed in 4% paraformaldehyde at room temperature for 15 min and washed with ice-cold phosphate buffered saline (PBS) for 5 min. To determine the stem or progenitor nature and confirm the endothelial lineage of cells, colonies were stained with anti-mouse CD-34-phycoerythrin, anti-mouse von Willebrandt factor (Santa Cruz Biotechnology, USA), anti-mouse VEGFR2-FITC (BD Biosciences, USA) and anti-mouse CD31-FITC (BioLegend, USA) antibodies for 1 h at 37°C. Cell nuclei were visualized using EverBrite mounting medium containing DAPI (Biotium, USA). Stained cells were examined by an AxioImager A1 fluorescent microscope (Carl Zeiss, Germany), images were acquired using a fluorescent camera and were documented with Zen2012 software (Carl Zeiss, Germany). Positively stained cells for markers were considered to be differentiated CEPs.

### In Vitro Mouse Blood-Brain Barrier Model

Isolation of primary MBECs from 3-month-old C57BL/6 mice were based on previously described methods from our group [31], [38], [39]. Mouse forebrains were removed from skulls and collected in ice-cold sterile PBS. Meninges and choroid plexuses were dissected, the tissue was cut to small pieces by scalpels and digested by collagenase (1 mg/ml; Worthington, USA) in Dulbecco’s modified Eagle medium (DMEM, Life Technologies, Gibco, USA) containing deoxyribonuclease type I (DNase I, Roche, Switzerland) at 37°C for 45 min. Cerebral microvessels were separated from myelin on a 20% BSA-DMEM gradient by centrifugation (1000×*g*, 20 min). This step was repeated three times. The collected vessel fractions were pooled and further digested by collagenase–dispase (1 mg/ml; Roche, USA) in DMEM containing DNase I for 35 min. The digested brain microvessel fragments were washed twice in cell culture medium then plated on Petri dishes (35 mm; Falcon; BD Biosciences, USA) coated with collagen type IV and fibronectin. MBECs were kept in DMEM/F12 supplemented with plasma-derived bovine serum (15%; First Link, UK), insulin (5 µg/ml), transferrin (5 µg/ml), sodium selenite (5 ng/ml), basic fibroblast growth factor (1 ng/ml; Roche, USA), heparin (100 µg/ml) and gentamycin (50 µg/ml). In the first 4 days cell culture medium contained puromycin (3 µg/ml) to selectively eliminate P-glycoprotein negative contaminating cell types [40]. When primary cultures reached 90% confluency on the fourth-fifth day after seeding, MBECs were passaged for experiments. Because of the low yield of primary brain endothelial cells from mice 24-well format culture inserts (ThinCert, 24-well format, polyethylene terephthalate membrane, 0.33 cm^2^ surface, 3 µm pore size, Greiner Bio-one, Germany) were selected, which were found to be optimal for BBB culture models [41]. For barrier integrity tests cells were subcultivated to the upper side of culture inserts at a cell number of 2.5×10^4^ cells/insert. Inserts were coated with collagen type IV and fibronectin. To enhance BBB characteristics, MBECs were co-cultured with primary mouse cerebral glial cells [32]. Primary cultures of mouse mixed glial cells were isolated from 1 or 2-day-old C57BL/6 mice [38], [39]. Meninges from brains were removed by fine forceps, then cortices were cut into small pieces and were mechanically dissociated by pressing the tissue through a nylon mesh (40 µm, Millipore, USA) in DMEM containing FBS (10%; Lonza, Switzerland) and gentamycin (50 µg/ml). Cell clusters were seeded on poly-l-lysine (5 µg/ml) coated 24-well plates (Greiner Bio-One, Germany) and cultured for two weeks before use. Confluent glia cultures contained 88% of cells stained for the astroglia cell marker glial fibrillary acidic protein (GFAP), and the other 12% were positive for CD11b, a microglia marker. To prepare the co-culture BBB model culture inserts were put into 24-well plates containing confluent primary mouse glia cultures at the bottom of the wells. MBECs were passaged to the upper side of the coated inserts and both compartments received endothelial culture medium [32]. After two days of co-culture cells were kept in medium containing 550 nM hydrocortisone. The day before the experiments cells were treated with chlorophenylthio-adenosine-3',5'-cyclic monophosphate (250 µM) and phosphodiesterase inhibitor RO 201724 (17.5 µM; Roche, USA) to strengthen junctions and increase resistance [40], [42].

### Measurement of Endothelial Monolayer Resistance and Permeability

One of the key parameters to validate BBB models is transendothelial electrical resistance (TEER). High TEER indicates low permeability of the paracellular pathway regulated by tight interendothelial junctions. TEER was measured by a Volt-Ohm resistance meter (World Precision Instruments, USA) using a chamber electrode and calculated relative to the surface of culture inserts (Ω×cm^2^). The TEER of empty, coated inserts (70 Ω×cm^2^) was subtracted from the measured values. On day 4 of co-culture cells were irradiated, and barrier functionality was tested 1, 2, 3 and 5 days after irradiation. The flux of fluorescein, a marker molecule for paracellular permeability and MRP-1 pump ligand (MW: 376 Da) and Evans blue-labeled albumin (MW: 67 kDa), marker for transendothelial permeability, was measured parallels across endothelial monolayers as previously described [39]. First TEER was measured, and then culture inserts were transferred to 24-well plates containing 530 µl Ringer-Hepes solution (118 mM NaCl, 4.8 mM KCl, 2.5 mM CaCl_2_, 1.2 mM MgSO_4_, 5.5 mM d-glucose, and 10 mM Hepes, pH 7.4). In the apical chambers culture medium was replaced by 70 µl Ringer-Hepes solution containing both 10 µg/ml fluorescein and 165 µg/ml Evans blue bound to 1% BSA. Plates containing the inserts were incubated on a horizontal shaker (100 rpm; Biosan, Latvia) at 37°C for 1 h. At 20, 40 and 60 min of the permeability assay culture inserts were placed to a new well containing Ringer-Hepes buffer. Samples from the luminal and abluminal compartments were collected. Evans blue-albumin content of samples was measured at 584 nm excitation and 680 nm emission wavelenghts (Fluostar Optima, BMG Labtechnologies, Germany). Fluorescein concentrations were determined by the same instrument using 485 nm excitation and 520 nm emission wavelenghts. Permeability across cell-free inserts for both marker molecules was also measured. Transendothelial permeability coefficient (Pe) was calculated as previously described [38], [39]. Clearance was expressed as µl of donor (luminal) compartment volume from which the tracer is completely cleared. Cleared volume was calculated from the concentration (C) of the tracer in the abluminal and luminal compartments and the volume (V) of the abluminal compartment (530 µl) by the following equation: Cleared volume (µl)  =  C*_abluminal_*×V*_abluminal_*/C*_luminal_*.

The average cleared volume was plotted *vs*. time, and permeability-surface area product (PS) values for endothelial monolayers (PSe) were calculated by the following equation: 1/*PS_endothelial_* = 1/*PS_total_*-1/*PS_insert_*.

PSe divided by the surface area (0.33 cm^2^) generated the endothelial permability coefficient (Pe; in 10^−6^ cm/s).

### Immunostainings and Quantification

Morphological changes in MBECs were investigated by immunohistochemical staining for claudin-5, zonula occludens protein 1 (ZO-1) and β-catenin. Confluent cultures of MBECs grown on collagen coated borosilicate glass coverslips (VWR, USA) were irradiated. After the respective postirradiation time cells were fixed with cold acetone-methanol (1∶1) for 10 min, washed with PBS and non-specific binding sites were blocked with 3% BSA-PBS for 1 h at room temperature. Incubation with anti-claudin-5, anti-ZO-1 (Life Technologies, Invitrogen, USA) and anti-β-catenin primary antibodies lasted overnight at 4°C. Cells were incubated with anti-rabbit secondary antibodies labeled with CY3 or Alexa Fluor 488 (Life Technologies, USA) and H33343 dye to stain nuclei for 1 h. Between each incubations cells were washed three times with PBS. Coverslips were mounted in Fluoromount-G (Southern Biotech, USA). Astroglia cells co-cultured and irradiated with endothelial cells were stained with mouse anti-GFAP primary antibody, anti-mouse Alexa 488 (Life Technologies, USA) secondary antibody and H33343 dye. Stainings were visualized by a Leica TCS SP5 confocal laser scanning microscope (Leica Microsystems, Germany). At least 5 non-overlapping pictures were taken for each stainings for each treatment groups. Cells were counted on immunohistochemical pictures and expressed as number of cells/mm^2^.

Image analysis was performed by using ImageJ, public domain software developed by the National Institutes of Health (USA). Digital images (512×512 pixels, n = 12–16 for each irradiation dose and time point) from different non-overlapping areas representing the total culture surface were captured for all 4 types of immunostainings. Immunostained areas were identified using the threshold feature of ImageJ. After subtracting the background fluorescence (subtraction of gray values of non-expressing pixels from every pixel) in the respective channel mean grayscale value was calculated and averaged.

### Senescence-Associated β-Galactosidase Staining

MBECs (1×10^4^) were grown on collage type IV and fibronectin coated glass coverslips in 6-well plates. To verify senescence, *in situ* staining for senescence-associated β-galactosidase (SA-β-gal) was performed [43]. Briefly, cells were washed with PBS and fixed with 2% formaldehyde and 0.2% glutaraldehyde in PBS for 5 min. After a PBS wash cells were incubated with β-galactosidase staining solution (150 mM NaCl, 2 mM MgCl_2_, 5 mM potassium ferricyanide, 5 mM potassium ferrocyanide, 40 mM citric acid, 12 mM sodium phosphate, pH 6.0 containing 1 mg/ml 5-bromo-4-chloro-3-indolyl-β-d-galactoside) for 24 h at 37°C. Stainings were analyzed under a light microscope; 400–500 cells/coverslip were counted and the percentage of senescent cells identified by blue staining was calculated.

### Statistical Analysis

Data are presented as means ± SD. Statistical significance between treatment groups was determined using one-way and two-way ANOVA following Bonferroni multiple comparison posttest (GraphPad Prism 5.0; GraphPad Software, USA). Changes were considered statistically significant at *p*<0.05 (*); *p*<0.01 (**) and *p*<0.001 (***). All experiments were repeated at least two times, the number of parallel samples varied between 3 and 16.

## Results

### Effect of Single Local Head Irradiation on Blood-Brain Barrier Permeability

**Figure 1:**
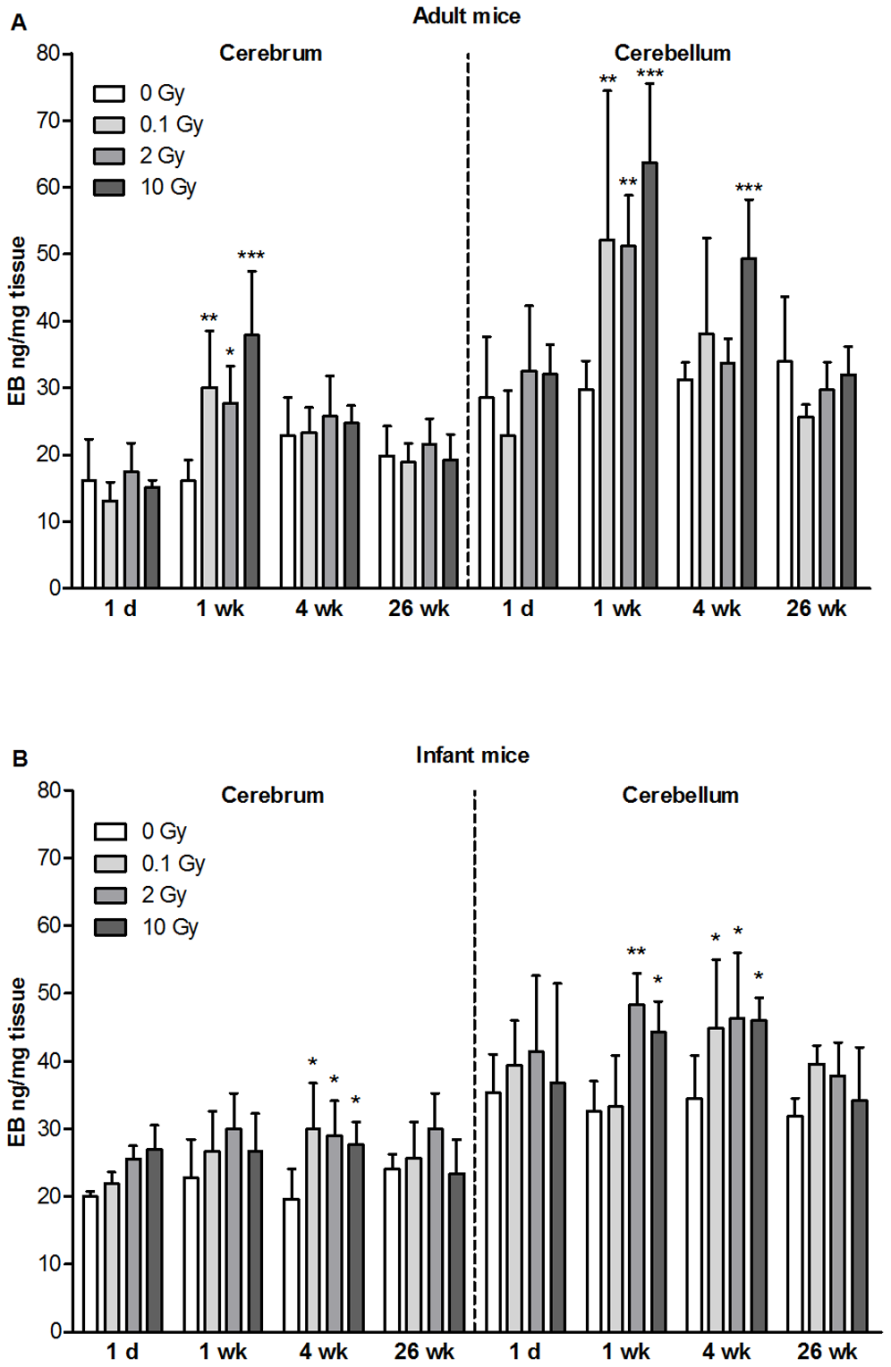
Effects of cranial irradiation on blood-brain barrier permeability in mice. Single head irradiation with 0.1, 2 and 10 Gy doses was performed on 10-week-old adult (A) and 10-day-old infant mice (B). BBB permeability for albumin was measured 1 day, 1, 4 and 26 weeks postirradiation and expressed as ng of Evans blue/mg of tissue. Values presented are means ± SD, n = 5–12 from 2–3 separate experiments. Statistical analysis: two-way ANOVA followed by Bonferroni post-test. Statistically significant differences *p*<0.05 (*); *p*<0.01 (**) and *p*<0.001 (***) are indicated compared to sham treated animals examined at the same time point. EB: Evans blue.

Brain vessel permeability of adult animals for albumin increased by about two-fold for all applied X-ray doses in both cerebrum and cerebellum 1 week after irradiation (Figure 1A). At 4 weeks postirradiation significant elevation was detected only at the highest dose in cerebellum. No change in albumin extravasation was seen at any further investigated time points in control and local head irradiated mice. In infant mice a significant increase in permeability was seen at 2 and 10 Gy in the cerebellum 1 week after X-ray exposure. The most prominent effect was an elevation of permeability in both brain regions in all treatment groups at 1 month after exposure, which was delayed in time compared to adult animals. No significant change in BBB permeability was detected in any of the irradiation and age groups 26 weeks after treatment, indicating a full recovery (Figure 1B).

### Effect of Head Irradiation on Body Weight

**Figure 2:**
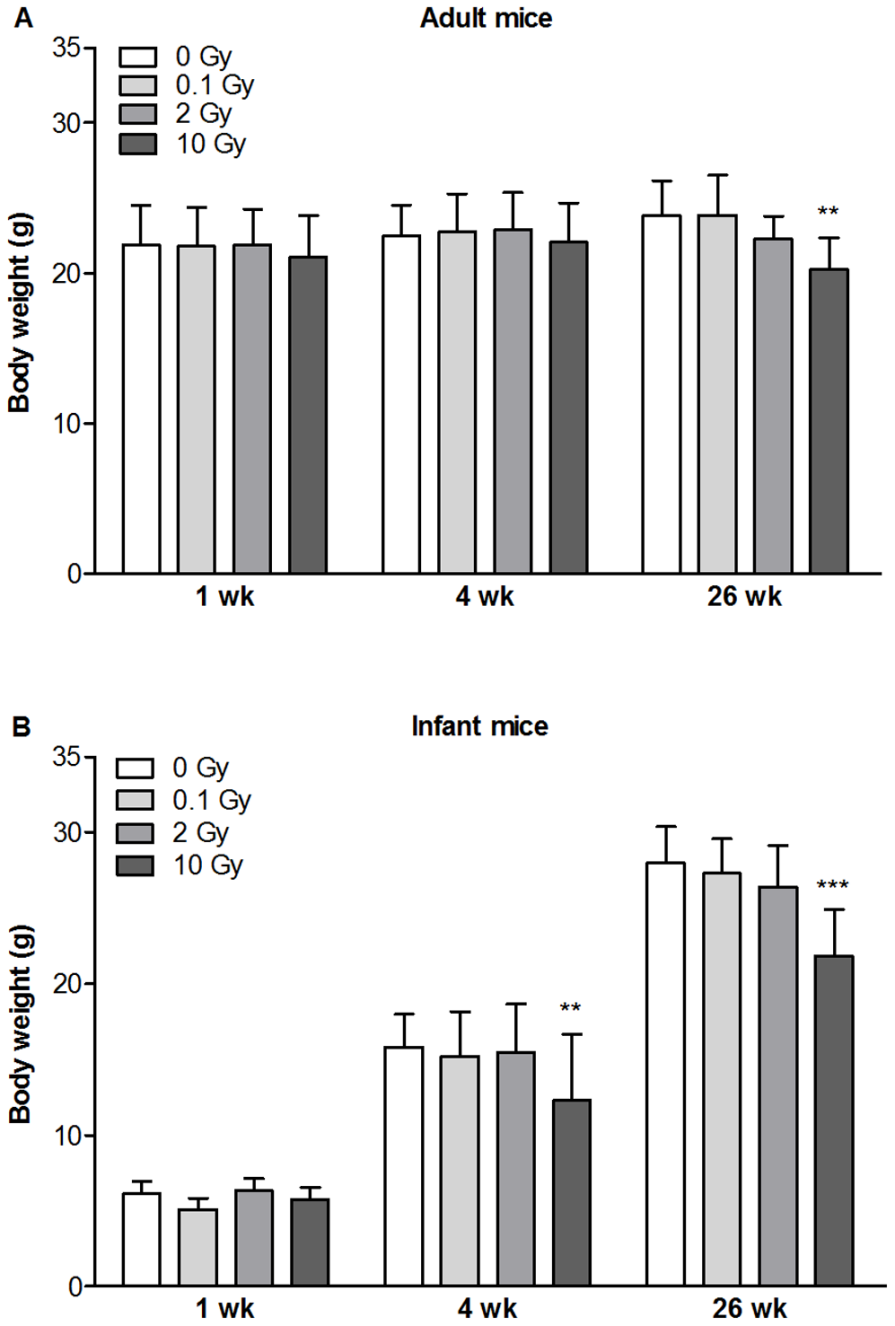
Effects of cranial irradiation on body weight in mice. Single head irradiation with 0.1, 2 and 10 Gy doses was performed on 10-week-old adult (A) and 10-day-old infant mice (B). Body weights were measured 1, 4 and 26 weeks postirradiation. Values presented are means ± SD, n = 8–32 from 4–6 separate experiments. Statistical analysis: two-way ANOVA followed by Bonferroni post-test. Statistically significant differences *p*<0.01 (**) and *p*<0.001 (***) are indicated compared to sham treated animals examined at the same time point.

Body weights were measured 1, 4 and 26 weeks after irradiation. Weight loss occurred only in mice exposed to 10 Gy in the adult group 26 weeks after local head irradiation (Figure 2A). Similarly, in infant mice only irradiation at 10 Gy decreased significantly body weight as compared to age-matched controls at 4 and 26 weeks (Figure 2B) indicating a strong biological effect of high dose head irradiation.

### Effect of Head Irradiation on Circulating Endothelial Progenitor Cell Number

**Figure 3:**
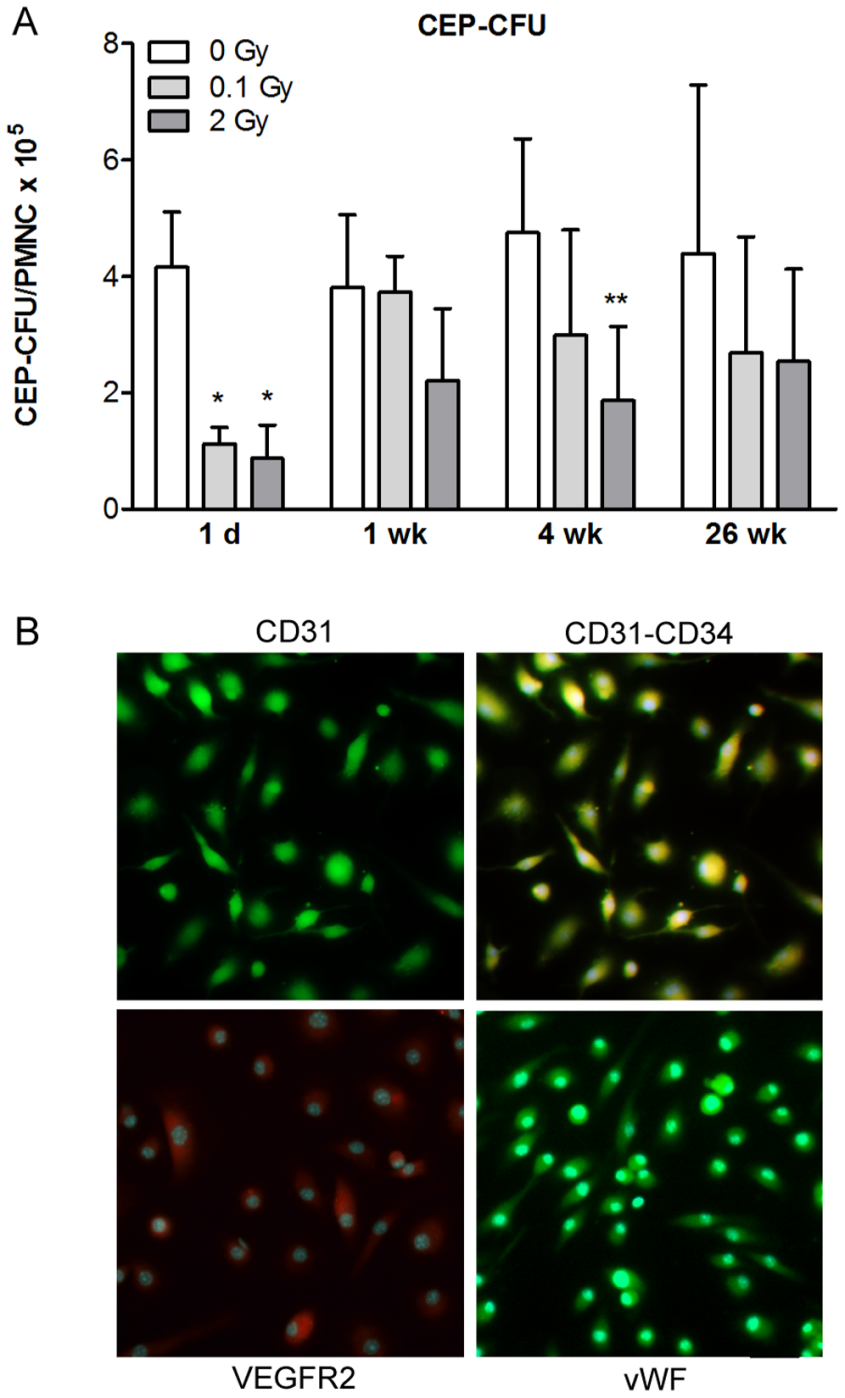
Effect of single dose cranial irradiation on circulating endothelial progenitor cell number. Single head irradiation with 0.1, 2 and 10 Gy doses was performed on 10-week-old adult mice. Circulating endothelial progenitor cell were isolated and cultured from peripheral blood mononuclear cells 1 day, 1, 4 and 26 weeks postirradiation (A). The number of endothelial progenitor cells is presented as colony forming unit/peripheral mononuclear cell ×10^5^/ml blood. Values presented are means ± SD, n = 3–10 from 2–3 separate experiments. Statistical analysis: two-way ANOVA followed by Bonferroni post-test. Statistically significant differences *p*<0.05 (*) and *p*<0.01 (**) are indicated compared to sham treated animals examined at the same time point. Representative images of endothelial progenitor cells immunostained for markers are presented in the panels (B): single staining for CD31; double labeling for CD31 and CD34; staining for VEGFR and vWF. CEP-CFU: circulating endothelial progenitor cell-colony forming unit; PMNC: peripheral blood mononuclear cell; CD31; cluster of differentiation 31, CD34; cluster of differentiation 34, VEGFR: vascular endothelial growth factor receptor, vWF: von Willebrand factor. Scale bar: 50 µm.

Blood-derived mouse CEPs were isolated 1 day, 1, 4 and 26 weeks after cranial X-ray exposure and cultured for 1–2 weeks. CEPs, derived from adherent PBMCs formed colonies surrounded by spindle-shaped cells, an early endothelial progenitor phenotype [44], [45], and gradually differentiated to a more endothelial-like morphology. Colonies of cultured CEPs stained positively for both CD34 and CD31 or vWF and VEGFR2 (Figure 3A, B) were counted. The number of CEPs colonies from cranial irradiated mice was significantly decreased 1 day after treatment and was still reduced at 4 weeks after 2 Gy exposure and gradually recovered by 26 weeks (Figure 3A). Cranial irradiation did not result in a significant drop in the number of PBMCs at day 1 (control: 15.53±2.43 10^5^ cells/ml; 0.1 Gy: 9.81±1.88 10^5^ cells/ml, *p*<0.071; 2 Gy: 9.99±1.41 10^5^ cells/ml, *p*<0.065; One way ANOVA + Bonferroni test) or any other time points.

### Effect of Irradiation on Barrier Integrity of Mouse Brain Endothelial Cells in Co-Culture

**Figure 4:**
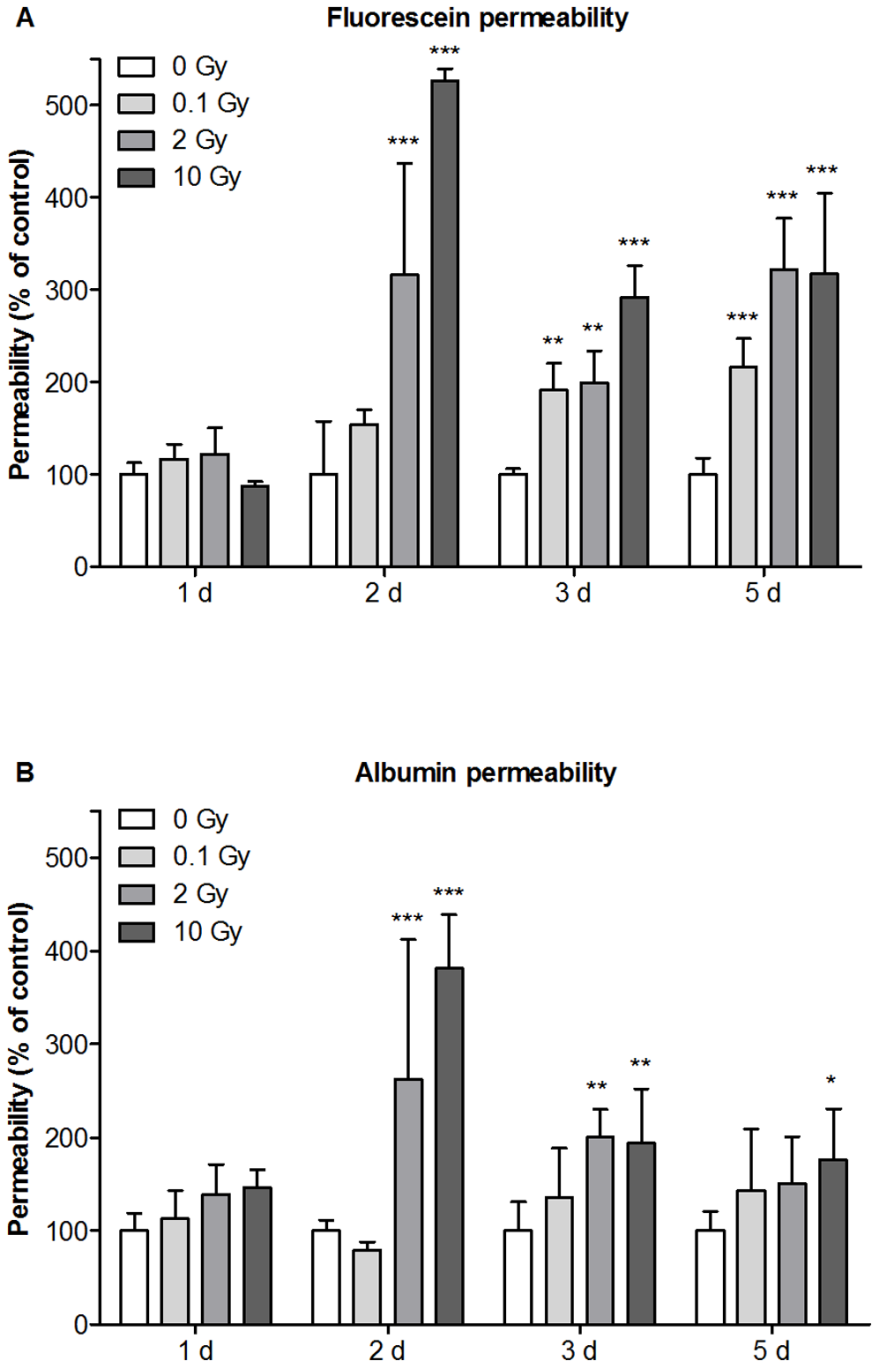
Effect of irradiation on mouse brain endothelial cell permeability. Primary mouse brain endothelial cells co-cultured with primary mouse glial cells were irradiated with a single dose of 0.1, 2 or 10 Gy. Permeability of mouse brain endothelial cell layers for fluorescein (A) and Evans blue labeled albumin (B) were measured 1, 2, 3 and 5 days postirradiation and expressed as percentage of the sham treated control. Values presented are means ± SD, n = 3. Statistical analysis: two-way ANOVA followed by Bonferroni post-test. Statistically significant differences *p*<0.05 (*); *p*<0.01 (**) and *p*<0.001 (***) are indicated.

The *in vitro* BBB model made from primary MBECs co-cultured with primary mouse glial cells showed good barrier properties reaching a TEER of 382.6±80.4 Ω×cm^2^ (n = 48). The tight barrier was also mirrored in the very low basal endothelial permeability coefficients for fluorescein (Pe = 0.21±0.09×10^−6^ cm/s) and albumin (Pe = 0.043±0.023×10^−6^ cm/s). No significant TEER changes were seen as compared to the control group in any of the treatment groups at any of the time points except for a 30% decrease at 10 Gy at day 2. At day 1 no change was seen in the permeability of marker molecules between the irradiation groups. High dose of irradiation (2 and 10 Gy) induced significant increase in permeability at days 2, 3 and 5. Single low dose exposure (0.1 Gy) had no effect at day 1 and 2, but a delayed elevation in fluorescein permeability was observed at days 3 and 5 (Figure 4A). Similarly to fluorescein flux, no change was detected in albumin permeability at day 1. Albumin penetration was significantly increased by irradiation with 2 and 10 Gy at days 2, 3 and 5. The biggest increase in permeability was seen at day 2 for both markers. In contrast to fluorescein, low dose exposure did not cause any effect on transcellular marker molecule penetration (Figure 4B).

### Effect of Irradiation on Morphology and Cell Number of Primary Mouse Brain Endothelial and Astroglia Cells

**Figure 5:**
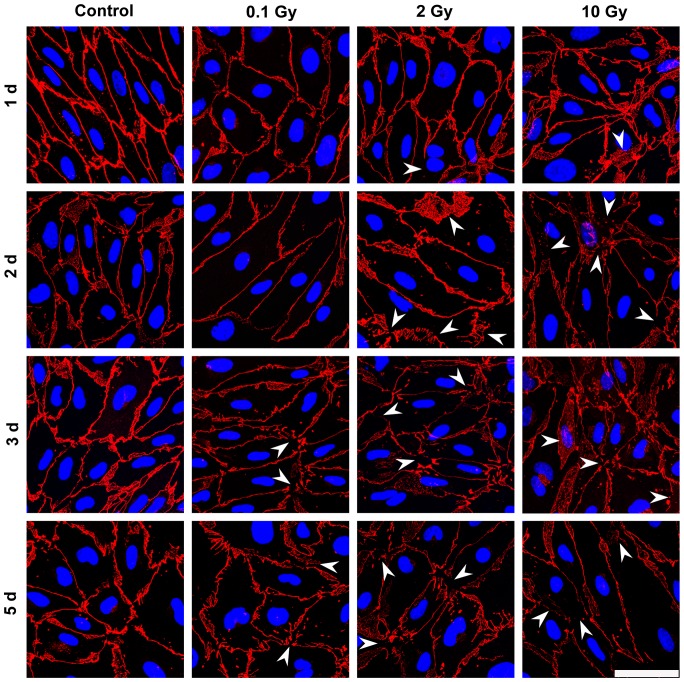
Effect of irradiation on claudin-5 immunostaining in mouse brain endothelial cells. Primary mouse brain endothelial cells 1, 2, 3 and 5 days after exposure to a single dose of 0.1, 2 or 10 Gy irradiation were immunostained for tight junction protein claudin-5. Arrowheads: fragmented junctional staining, gap between cells or cytoplasmic redistribution of the junctional protein. Red color: immunostaining for claudin-5. Blue color: H33343 staining of cell nuclei. Bar  = 50 µm.

**Figure 6:**
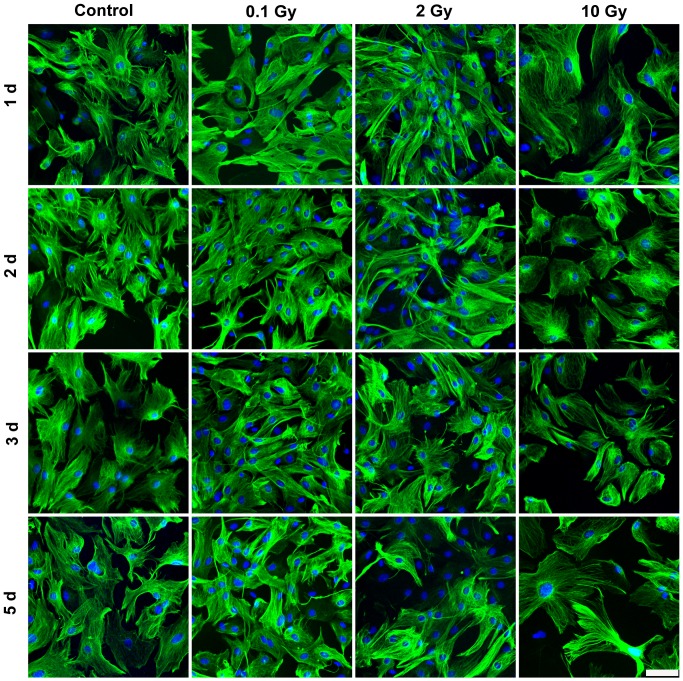
Effect of irradiation on GFAP immunostaining in mouse astroglia cells. Primary mouse astroglia cells 1, 2, 3 and 5 days after exposure to single dose of 0.1, 2 or 10 Gy irradiation were immunostained for glial fibrillary acidic protein (GFAP). Green color: immunostaining for GFAP. Blue color: H33343 staining of cell nuclei Bar  = 50 µm.

**Figure 7:**
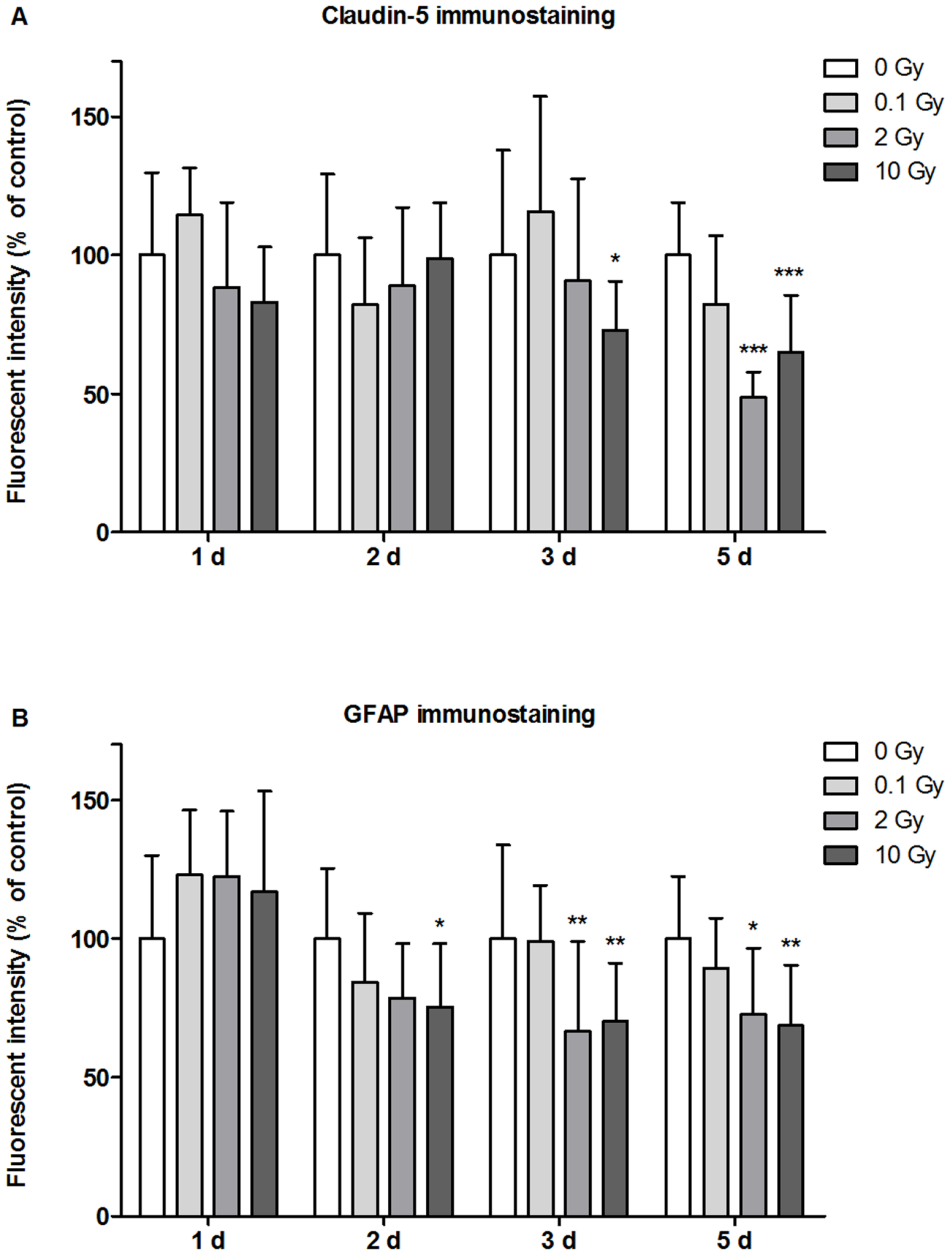
Quantification of Claudin-5 and GFAP immunostaining by image analysis. Fluorescent intensity of claudin-5 immunostaining in primary mouse brain endothelial cells (A) and GFAP staining in primary mouse astroglia cells (B) was evaluated 1, 2, 3 and 5 days after exposure to single dose of 0.1, 2 or 10 Gy irradiation using ImageJ software. Values presented are means ± SD, n = 12–16. Statistical analysis: two-way ANOVA followed by Bonferroni post-test.

In MBECs the effect of irradiation on cell-cell adhesion and morphology was investigated by immunostaining for transmembrane tight junction protein claudin-5 (Figure 5), and cytoplasmic linker proteins β-catenin and ZO-1 (Figure S1–S2). In the control group all three junctional stainings were strong, and localized to the cell borders. The elongated shapes of endothelial cells were well delineated by the belt-like staining. Cell-cell attachment in MBECs was continuous, without gaps. No gross changes in junctional immunostainings were visible 24 h post-irradiation. Interendothelial junctional staining was changed in treated groups 2, 3 and 5 days post-irradiation, which effect was also reflected by permeability elevation at these time points. The pattern of the staining was especially altered at 3 and 5 days at 2 and 10 Gy: intercellular gaps, fragmented junctional staining and cytoplasmic redistribution of junctional proteins were observed (Figure 5). Morphological changes in astroglia cells were monitored by GFAP staining which revealed polygonal shape and several long processes in the sham irradiated group, typical for cultured type 1 astrocytes (Figure 6). This stellate shape was also observed in the 0.1 and 2 Gy groups, although more bipolar cells were present. The loss of polygonal shape and fine processes were more pronounced in cells exposed to 2 Gy irradiation. Striking morphological changes were visible in astrocytes irradiated with 10 Gy at all time points. A decreased intensity of the fluorescent immunostaining for junctional proteins was measured by quantitative image analysis in the moderate and high dose irradiation groups 3 and 5 days after treatment (Figure 7A, S3). In the high dose irradiation group at days 3 and 5 flattened morphology and increase of cell surface indicating cell stretching was prominent in MBECs (Figure 5, S1, S2). Changes in GFAP immunostaining in glial cells were quantified by image analysis, and significant decreases in intensity were observed at 2 and 10 Gy treatment doses on day 2, 3 and 5 postirradiation (Figure 7B).

**Figure 8:**
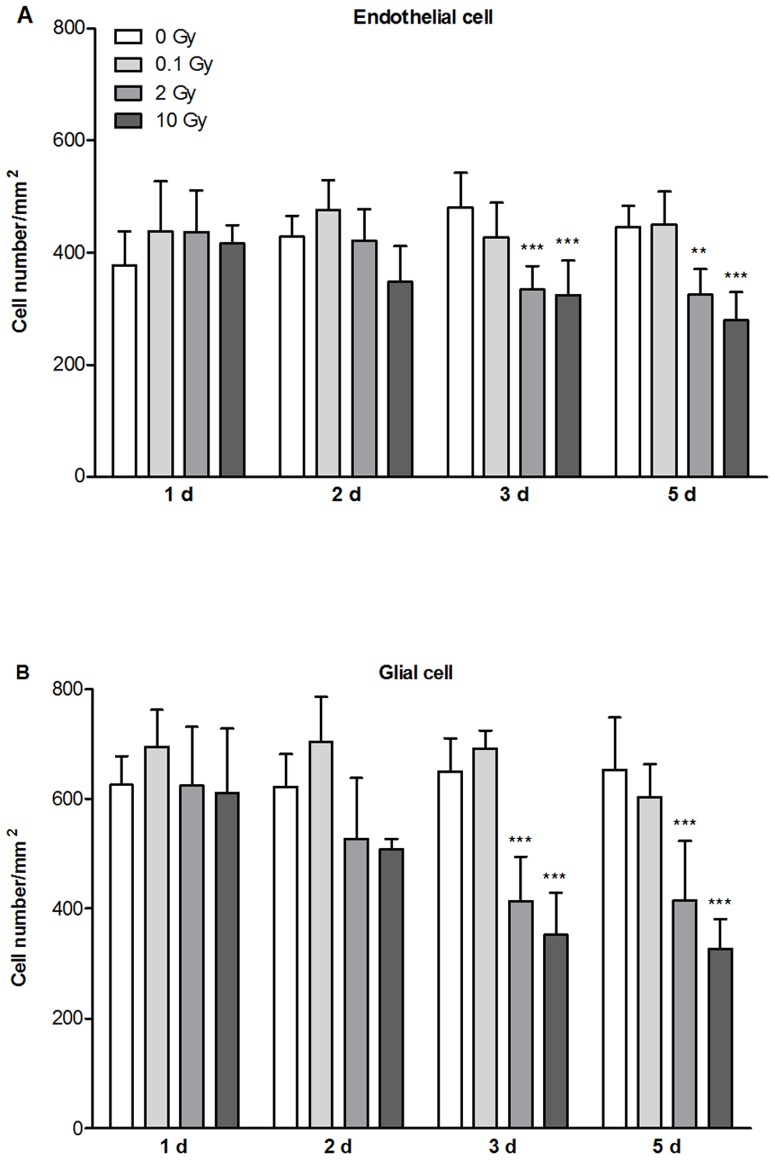
Effect of irradiation on the number of primary mouse brain endothelial and astroglia cells. Effect of irradiation was measured on the number of primary mouse brain endothelial (A) and astroglia (B) cells 1, 2, 3 and 5 days after exposure to single dose of 0.1, 2 or 10 Gy irradiation. Values presented are means ± SD, n = 5–6 from 2 separate experiments. Data is expressed as number of cells/mm^2^. Statistical analysis: two-way ANOVA followed by Bonferroni post-test. Statistically significant differences *p*<0.01 (**) and *p*<0.001 (***) are indicated.

No change in MBEC and astroglia cell number was seen at day 1 and 2 (Figure 8). Low dose of irradiation (0.1 Gy) had no effect on endothelial or astroglia cell number. A significant decrease in cell number for both cell types was detected at the two highest doses of irradiation on days 3 and 5 (Figure 8). This decrease in cell number is clearly visible on the immunohistochemical stainings (Figure 5, 6, S1, S2).

### Characterization of Irradiation-induced Injury in Cultured Primary Mouse Brain Endothelial Cells

**Figure 9:**
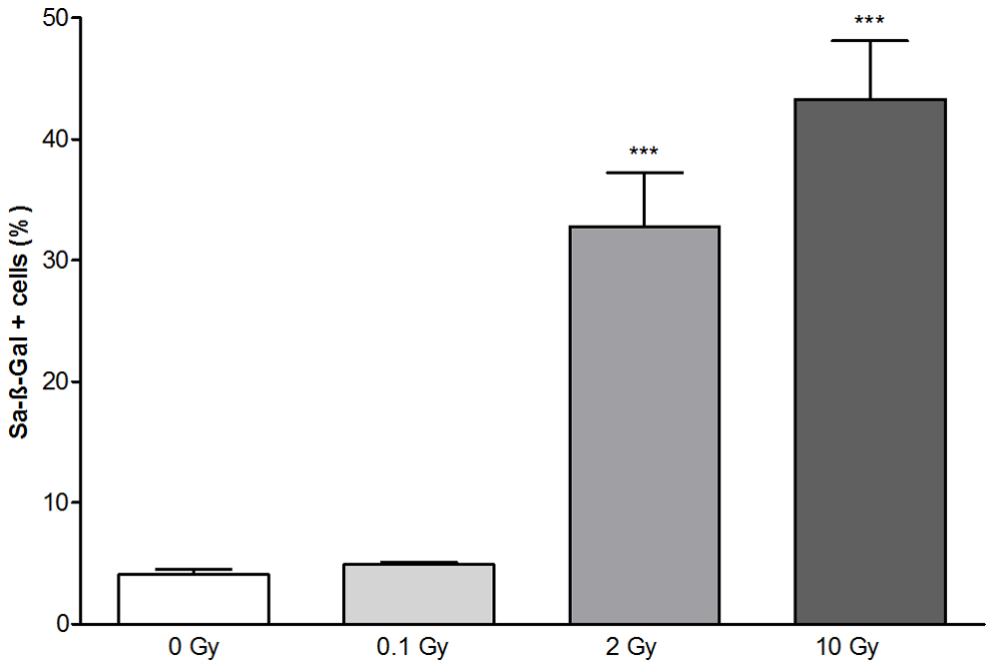
Effect of irradiation on senescence of mouse brain endothelial cells. Ratio of senescence associated-β-galactosidase positive primary mouse brain endothelial cells was determined 5 days after exposure to a single dose of 0, 0.1, 2 or 10 Gy irradiation. Values presented are means ± SD, n = 19 from 2 separate experiments. Data is expressed as percentage of positively stained cells. Statistical analysis: one-way ANOVA followed by Bonferroni post-test. Statistically significant difference *p*<0.001 (***) is indicated.

Considering that regenerative capacity contributes greatly to endothelial function, we further examined the radiation-induced dose dependency of cellular senescence in MBECs. SA-β-gal staining, a characteristic feature of senescent cells [43] was tested in irradiated MBECs. The percentage of SA-β-gal-positive cells did not change in the 0.1 Gy irradiation group, but significantly increased by 30 and 40% at 2 and 10 Gy exposure as compared to unirradiated controls at day 5 (Figure 9). A flow cytometric method (Text S1) was used to analyze the repair kinetics of double strand DNA breaks in irradiated MBECs. Irradiation induced a dose-dependent increase in the phosphorylation of H2A.X at 10 min (Figure S4). In 0.1 and 2 Gy irradiation group repair was complete by 1 h posttreatment. In the 10 Gy irradiation group phosphorylation of H2A.X was still elevated not only at 1 and 4 h, but remained higher than the baseline level by 24 h indicating impaired repair capacity in MBECs.

## Discussion

The risk of cerebrovascular diseases is increased and the integrity of BBB is impaired by X-ray radiation especially after high doses. The effect of low dose (≤100 mGy) ionizing radiation is debated, and its risk in clinical setting is currently reevaluated, but there are only few reports describing functional or morphological changes caused by low dose irradiation. Our hypothesis was that low dose cranial irradiation damaged BBB integrity and endothelial repair from circulating progenitors. We supposed that cell aging may be one of the mechanisms of the radiation-induced brain endothelial injury. In the present study, we wanted to clarify using *in vivo* and *in vitro* model systems (i) the effect of low dose X-ray radiation on BBB damage, (ii) the age dependence of irradiation-induced BBB injury, (iii) the effect of cranial irradiation on circulating endothelial progenitors and (iv) reversibility of radiation-induced effects at the level of the BBB.

This is the first study to show that not only moderate and high doses of irradiation with 2 and 10 Gy, but a single low dose cranial irradiation with 0.1 Gy also induced BBB injury in adult mice. In our experiments the peak of BBB permeability for albumin was 1 week after irradiation. No dose-dependency was observed at 1 week, but 4 weeks after radiation permeability increase was seen at the highest 10 Gy dose in the cerebellum. Radiation-induced BBB breakdown is unequivocally demonstrated at high doses [46]. Clinically relevant cumulative high doses can reach 40–60 Gy in patients with malignant brain tumors [14], [47]–[49], and both early BBB disruption and late effects are observed [13], [50]. In case of accidental exposure, the relative risk of fatal stroke is increased (0.12, 90% CI: 0.02–0.22) for the dose of 1 Gy in Hiroshima and Nagasaki survivors [51]. In rats 20 and 40 Gy brain irradiation produces an early increase in BBB permeability [22]. A time- and dose-dependent loss of brain microvessels and decrease in vessel growth in the hippocampus of mice can be observed as long as 12 months after a single exposure to ^56^Fe ions at doses of 0.5, 2 and 4 Gy [52]. The lowest irradiation dose studied so far is 4.5 Gy total body irradiation which induces early BBB permeability increase in rats [53]. Our findings suggest that BBB is sensitive even to single low dose head irradiation. In the present mouse study the 1-day and 1-week observation time points can be correlated to acute effects, the 1-month time point to early delayed, the 6-month time point to delayed late radiation effect (1). While we observed acute and early delayed effects, we did not observe a delayed late BBB impairment in either long-term followed group (6-month). Delayed late radiation-induced brain injury, characterized by vascular abnormalities, edema, demyelination, and white matter necrosis, is recognized as a dose-limiting morbidity evident more than six months after cranial radiotherapy in patients. In our study where doses were much lower than the therapeutic range, single low dose exposure did not cause late progressive BBB damage. Our findings draw attention to the fact that the acute effects of low doses can not be neglected.

In the present experiments cranial single low dose X-ray radiation also impaired the integrity of the BBB in infant mice, but the increase of permeability was delayed compared to adults. Moderate and high dose X-ray radiation with 2 and 10 Gy increased the permeability 1 week postirradiation in cerebellum of infant mice, but at 4 weeks all doses including 0.1 Gy elevated albumin extravasation in both brain regions. We can not exclude that in addition to the developmental age anesthesia with ketamine, an NMDA type glutamate receptor antagonist contributed to the difference in the radiation-induced BBB injury in infant and adult mice in our study. Cultured brain endothelial cells from young mice react on NMDA receptor modulators in a different way than brain endothelial cells from adult mouse brains [54]. Similarly to our data the BBB opening in young rats (4-week-old) irradiated with 20 and 40 Gy is already seen at 1 week postirradiation and remains open until 9 weeks [22]. Higher doses and species differences in radiosensitivity may contribute to the different kinetics. Importantly, the albumin leakage measured both in adult and infant mice after X-ray exposure was completely reversible by 4 and 26 weeks in adults and 26 weeks in infant mice. These results were consistent with a previous report [55], where BBB damage caused by 20 or 40 Gy was also reversible in adult rats. According to our and previous data [22] change in BBB permeability is an early and sensitive index for the detection of low dose radiation-induced endothelial damage.

Other biological effects of cranial irradiation were also observed in our experiments. Low and moderate doses of irradiation had no effect on body weight of infant and adult animals but 10 Gy X-ray exposure caused a significant weight loss in both age groups. Body weight was reduced at 4 and 26 weeks in infant mice indicating a prolonged effect most probably due to the injury of salivary glands resulting in feeding difficulties [56]. These data are in agreement with previous studies showing body weight loss and aging after high dose irradiation [57], [58].

Vascular repair and rapid reendothelization after injury are widely studied and long-time known [59]. Mechanisms of repair include migration, division and stretching of surviving endothelial cells adjacent to irradiated regions [60]. CEPs as stem cells play a key role in the maintenance of vascular integrity by replacing and repairing the damaged endothelial cells in the tissues [28] also after irradiation-induced vascular injury [16]. CEPs and hematopoietic stem cells are both derived from a common precursor [61] and CEPs possess both stem and endothelial cell surface markers including CD34, CD31, VEGFR2, vWF and CD133 [62], [63]. We successfully isolated CEPs from peripheral blood in mice expressing several of these markers. We investigated the number of mobilized CEPs in blood after local cranial irradiation which was reduced in the circulation at 1 day postirradiation. We assume that these circulating progenitor cells were eliminated from the blood by the partial body irradiation. Total body irradiation (6.5 Gy) in mice decreases hematopoietic stem cell life span [64]. In the present experiment much lower and local X-ray exposure had an effect on the number of CEPs from bone marrow derived progenitor cells indicating a sensitivity of circulating progenitors to low and moderate doses of irradiation. A reduced level of CEPs after low dose irradiation may contribute to impaired repair of the BBB and prolonged leakage of serum proteins.

To understand the cellular mechanisms of low, moderate and high dose irradiation on the BBB we examined the effects of X-ray exposure on an *in vitro* model developed and regularly used by our group [31]–[33], [39], [40]. Co-culture of primary MBEC with glial cells induced barrier properties and stainings for tight junction markers in endothelial cells, and the monolayers showed high TEER and low basal permeability similarly to the rat model [32], [33]. Endothelial monolayer permeability was dose- and time-dependent after X-ray exposure. Cultures were sensitive to irradiation, low doses also caused permeability elevation 3 and 5 days postirradiation. The increased permeability was accompanied by altered junctional immunostaining: intercellular gaps, fragmented junctional labeling and cytoplasmic redistribution of junctional proteins were observed similarly to the effect of other pathological agents on cultured brain endothelial cells [38], [65]. For the immunohistochemical labeling three kinds of junctional proteins were chosen: tight junction protein claudin-5, the main claudin in brain endothelial cells, its cytoplasmic linker protein ZO-1 and β-catenin, a cytoplasmic linker of adherens junction proteins. The main reason to select a linker and not a transmembrane adherens junction protein was that β-catenin is also a key member of the Wnt signaling pathway that controls the development and barrier function of the BBB [66]. In agreement with our results an increased and persistent monolayer leakage and zipper-like staining for junctional proteins is present in cultured bovine brain endothelial cells after irradiation with higher doses [26]. The mouse co-culture BBB model which can be kept for about a week without loss of permeability properties was useful to study single radiation treatment, but longer maintenance of the cultures is not possible which limits its application for the study of long-term effects or chronic fractioned radiation. The presence of glial cells induces barrier properties in physiological and influences changes in pathological conditions in cultured brain endothelial cells [67]. Primary endothelial cells were grown and treated on coverslips for immunostainings in mono-culture without receiving glial-cell conditioned medium, but these brain endothelial cells still showed typical monolayer morphology and junctional stainings, while changes in cell culture stainings on cover slips reflected well the radiation treatment effects seen in the permability experiments.

In addition to permeability and morphological changes a drop in cell density of both endothelial and glial cultures was seen with the two highest doses. In accordance with our results decrease in rat brain endothelial cell number [68], and glial cell loss in the spinal cord [69] occurs in vivo, and a significant decrease in brain endothelial cell density is seen in vitro after irradiation [26]. The integrity of the vascular endothelial layer is important and despite the drop in cell number brain endothelial cells maintain confluent layers even after injury resulting in a decreased cell density and increased cell size. Endothelial cell stretch and hypertrophy after irradiation participate in this phenomenon [70], [71].

To reveal how X-ray exposure impairs endothelial cell permeability, we investigated senescence under the same conditions *in vitro* permeability tests were performed. The number of SA-β-gal-positive senescent brain endothelial cells increased by 2 Gy and 10 Gy treatment, which is consistent with the findings of Lee et al. on human endothelial progenitor cells [16]. Endothelial senescence is linked to both aging and irradiation-induced vascular pathologies [72]–[74]. Decrease of basal endothelial nitric oxide levels, increase in reactive oxygen species and inflammation contribute to telomere dysfunction and replicative senescence of vascular endothelial cells [74], [75]. We hypothesize that senescence, cell number decrease and changes in tight junction morphology all participate in permeability elevation in brain endothelial cells.

Measurement of phosphorylation of histone H2A.X is a marker for the induction of double strand DNA breaks and toxicity following damage [76], [77]. We found that phosphorylation of H2A.X in brain endothelial cells increased with all tested irradiation doses including 0.1 Gy indicating radiation-induced DNA damage. Low dose irradiation also increases histone phosphorylation and DNA damage in human fibroblasts and endothelial cells [78], [79]. Importantly, low (0.1 Gy) and moderate dose (2 Gy) radiation-induced H2A.X phosphorylation returned to baseline in brain endothelial cells within 24 h indicating an effective repair mechanism.

Our study is the first to show that clinically relevant low dose irradiation induced BBB impairment. Not only high, but moderate and low dose of X-ray radiation caused albumin leakage in brain vessels of both adult and infant mice. In infant animals irradiation caused BBB permeability elevation was delayed compared to adult animals, but it was fully reversible with time in both age groups. The number of endothelial progenitor cells decreased after irradiation which may contribute to impaired repair of the BBB. Irradiation increased albumin permeability of monolayers in parallel with changes in intercellular junctional staining, drop in cell density and increase in the ratio of senescent cells in brain endothelial cultures. Low dose X-ray radiation caused DNA damage in brain endothelial cells, but an effective DNA repair mechanism was present. Low dose X-ray radiation caused impairment of BBB integrity both *in vivo* and *in vitro* supporting the need to reevaluate the risks of radiation exposure, especially at young age.

## Supporting Information

Figure S1 **Effect of irradiation on ZO-1 immunostaining in mouse brain endothelial cells.** Primary mouse brain endothelial cells 1, 2, 3 and 5 days after exposure to a single dose of 0.1, 2 or 10 Gy irradiation were immunostained for cytoplasmic junctional linker zonula occludens-1 protein. Arrowheads: fragmented junctional staining, gap between cells or cytoplasmic redistribution of the junctional protein. Green color: immunostaining for ZO-1. Blue color: H33343 staining of cell nuclei. Bar  = 50 µm.
(TIF)

Figure S2 **Effect of irradiation on β-catenin immunostaining in mouse brain endothelial cells.** Primary mouse brain endothelial cells 1, 2, 3 and 5 days after exposure to a single dose of 0.1, 2 or 10 Gy irradiation were immunostained for cytoplasmic junctional linker protein β-catenin. Arrowheads: fragmented junctional staining, gap between cells or cytoplasmic redistribution of the junctional protein. Red color: immunostaining for β-catenin. Blue color: H33343 staining of cell nuclei. Bar  = 50 µm.
(TIF)

Figure S3 **Quantification of ZO-1 and β-catenin immunostaining by image analysis.** Fluorescent intensity of ZO-1 (A) and β-catenin (B) immunostaining in primary mouse brain endothelial cells was evaluated 1, 2, 3 and 5 days after exposure to a single dose of 0.1, 2 or 10 Gy irradiation using ImageJ software. Values presented are means ± SD, n = 12. Statistical analysis: two-way ANOVA followed by Bonferroni post-test.
(TIF)

Figure S4 **Effect of irradiation on repair kinetics of double strand DNA breaks in irradiated mouse brain endothelial cells.** Fluorescence intensity of phosphorylated H2A.X immunostaining indicating DNA double strand breaks in mouse primary endothelial cells 10 min, 60 min, 4 h and 24 h after exposure to 0, 0.1, 2, 10 Gy irradiation. Values presented are means ± SD, n = 6 from 2 separate experiments. Statistical analysis: two-way ANOVA followed by Bonferroni post-test. Statistically significant difference *p*<0.001 (*) and *p*<0.001 (***) is indicated.
(TIF)

Text S1 (DOCX)
