# Supplementary figures and images for: Low Dose Cranial Irradiation-Induced Cerebrovascular Damage Is Reversible in Mice
Source: PLoS One. 2014 Nov 13;9(11):e112397. doi: 10.1371/journal.pone.0112397 (PMC4231057; doi:10.1371/journal.pone.0112397)

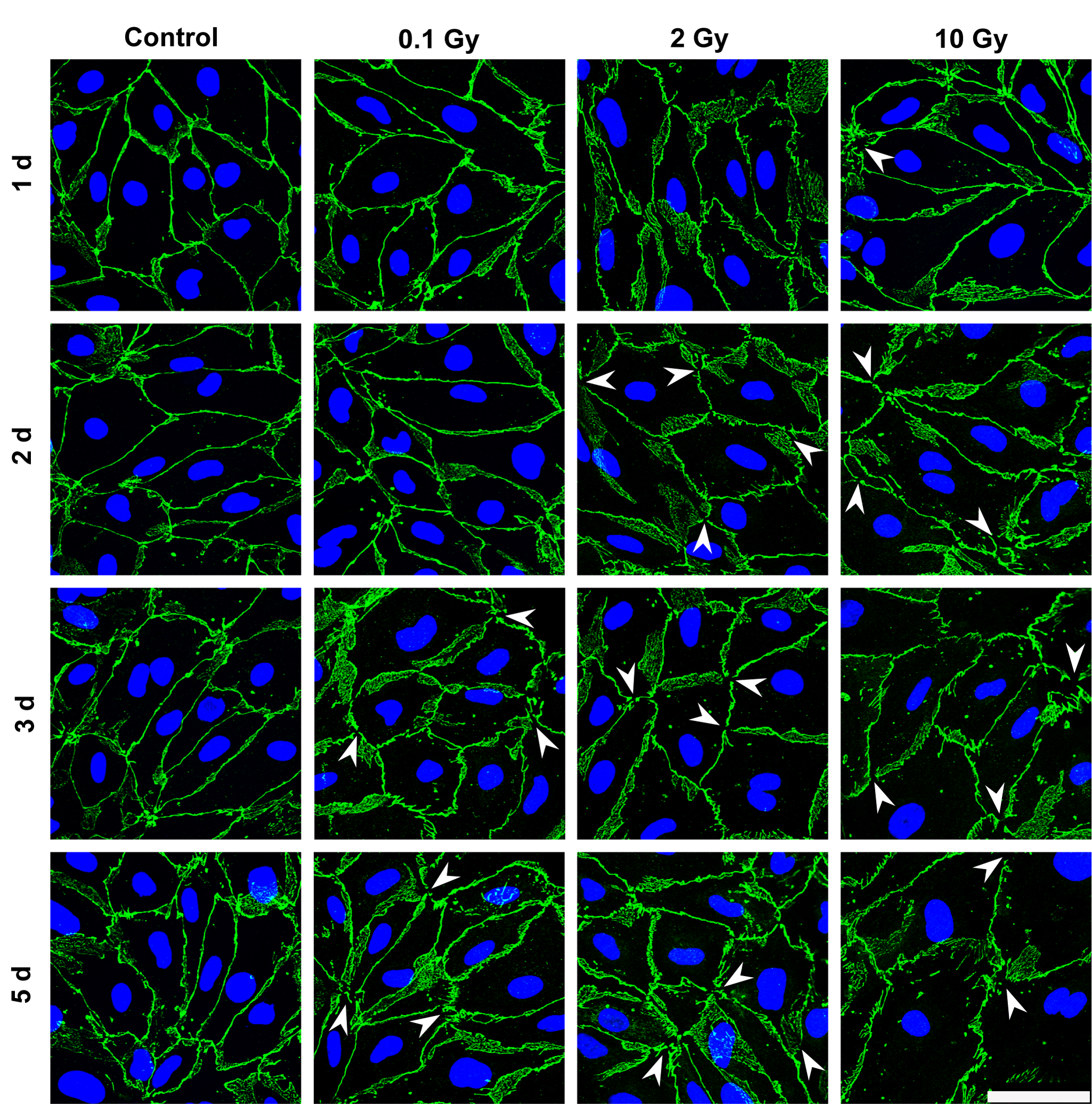

Supplement: Figure S1 — Effect of irradiation on ZO-1 immunostaining in mouse brain endothelial cells. Primary mouse brain endothelial cells 1, 2, 3 and 5 days after exposure to a single dose of 0.1, 2 or 10 Gy irradiation were immunostained for cytoplasmic junctional linker zonula occludens-1 protein. Arrowheads: fragmented junctional staining, gap between cells or cytoplasmic redistribution of the junctional protein. Green color: immunostaining for ZO-1. Blue color: H33343 staining of cell nuclei. Bar = 50 µm. (TIF) [file pone.0112397.s001.tif]

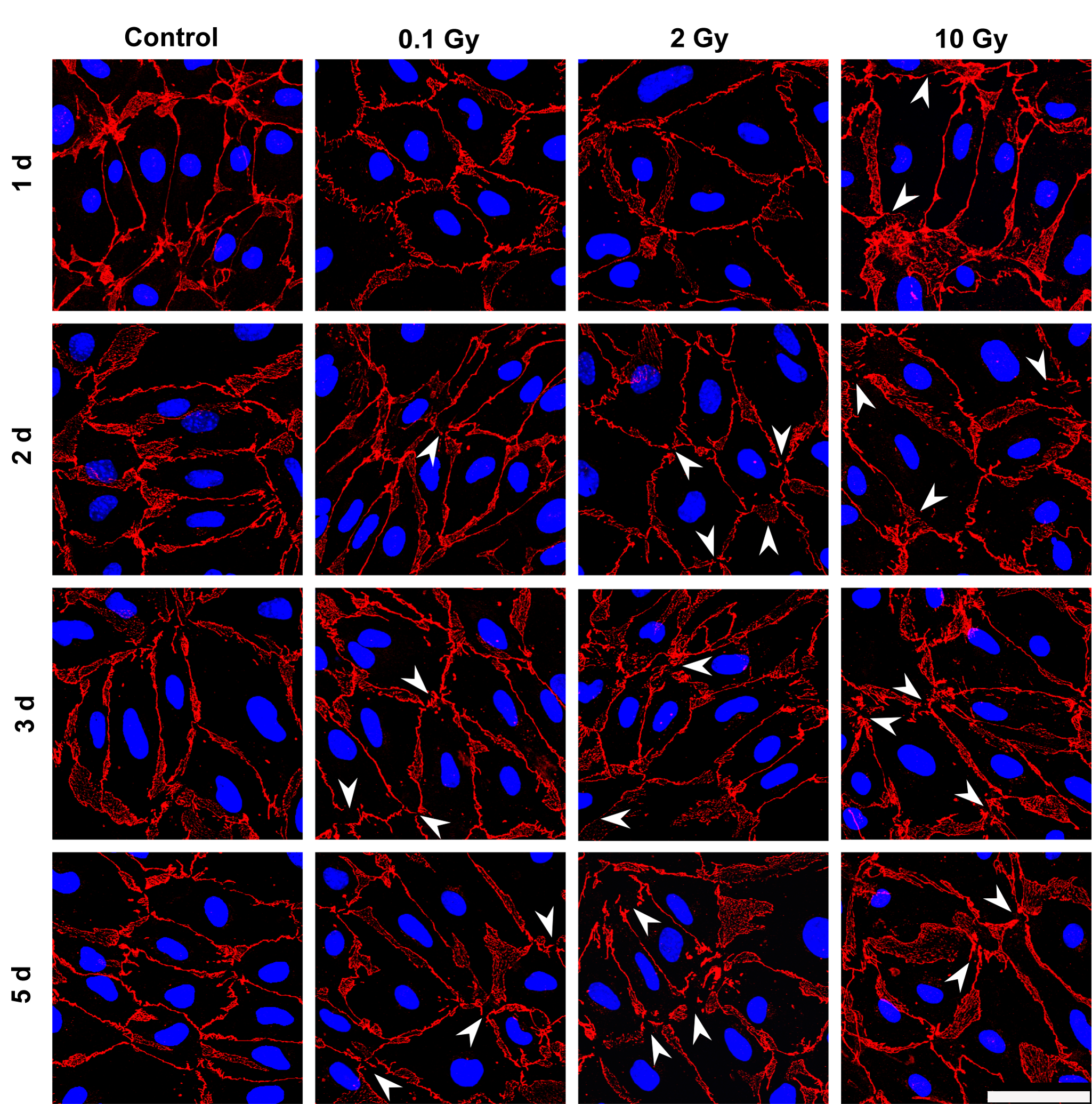

Supplement: Figure S2 — Effect of irradiation on β-catenin immunostaining in mouse brain endothelial cells. Primary mouse brain endothelial cells 1, 2, 3 and 5 days after exposure to a single dose of 0.1, 2 or 10 Gy irradiation were immunostained for cytoplasmic junctional linker protein β-catenin. Arrowheads: fragmented junctional staining, gap between cells or cytoplasmic redistribution of the junctional protein. Red color: immunostaining for β-catenin. Blue color: H33343 staining of cell nuclei. Bar = 50 µm. (TIF) [file pone.0112397.s002.tif]

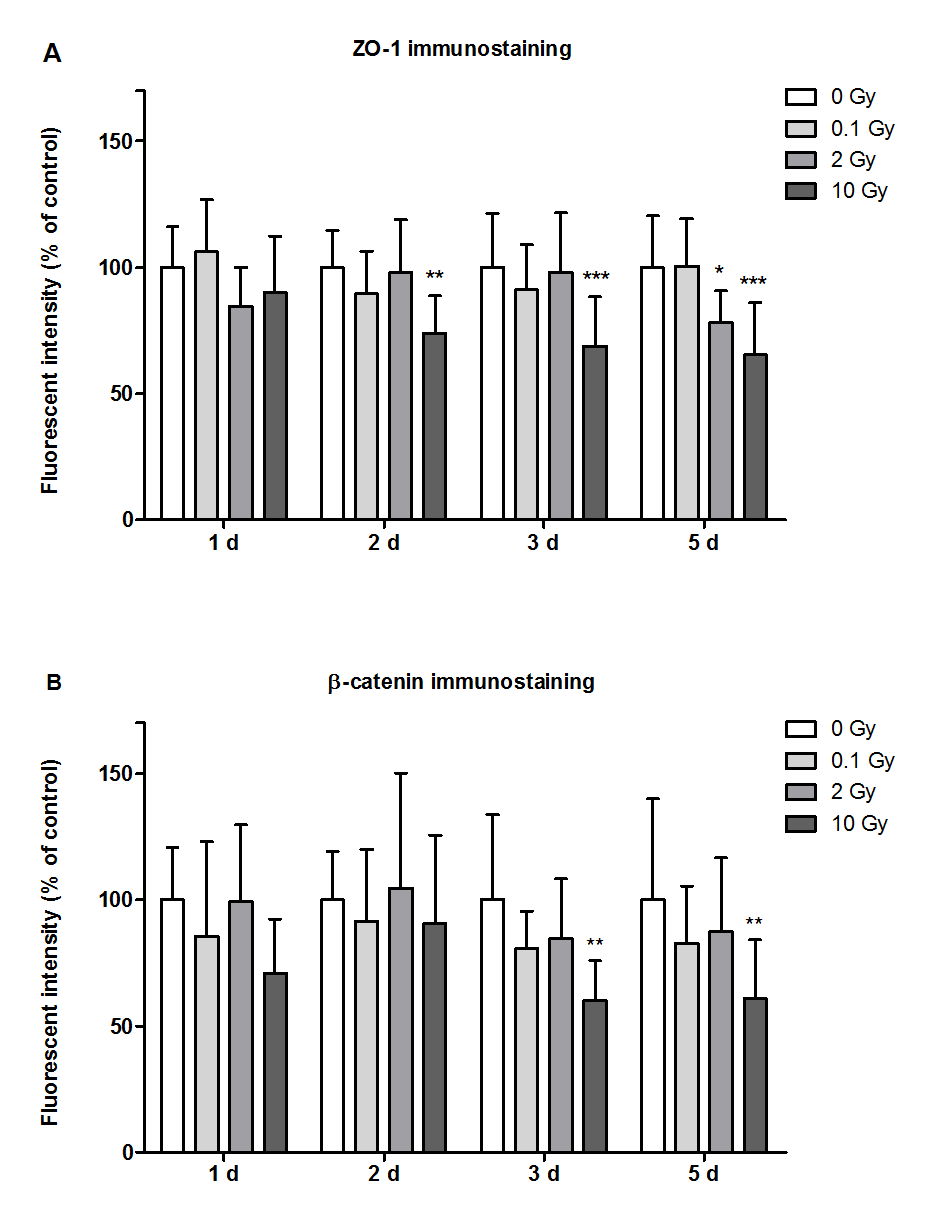

Supplement: Figure S3 — Quantification of ZO-1 and β-catenin immunostaining by image analysis. Fluorescent intensity of ZO-1 (A) and β-catenin (B) immunostaining in primary mouse brain endothelial cells was evaluated 1, 2, 3 and 5 days after exposure to a single dose of 0.1, 2 or 10 Gy irradiation using ImageJ software. Values presented are means ± SD, n = 12. Statistical analysis: two-way ANOVA followed by Bonferroni post-test. (TIF) [file pone.0112397.s003.tif]

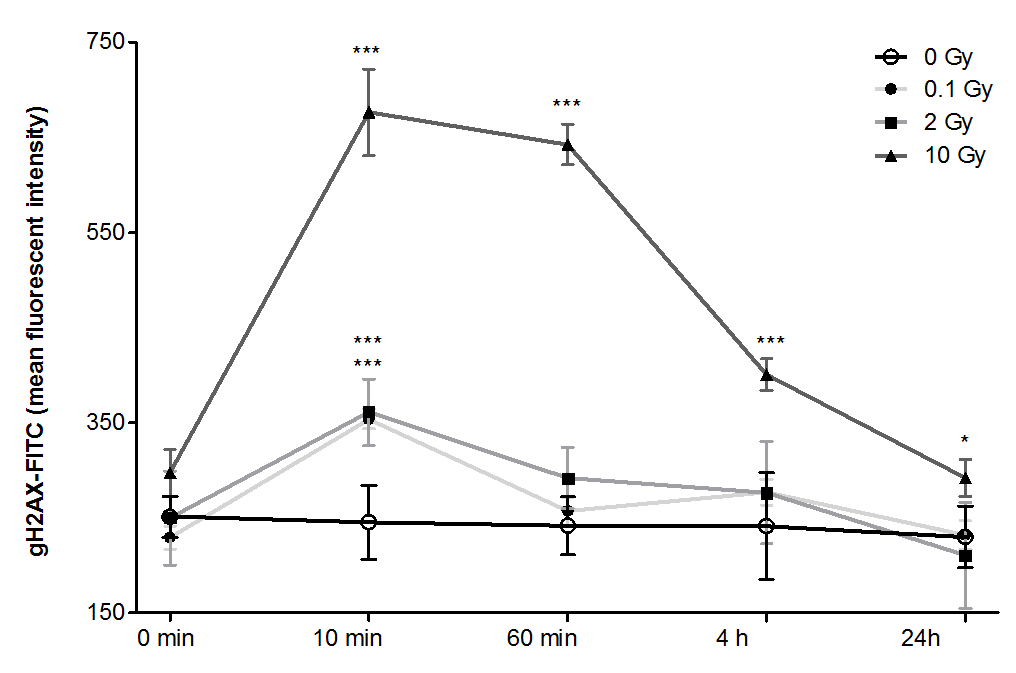

Supplement: Figure S4 — Effect of irradiation on repair kinetics of double strand DNA breaks in irradiated mouse brain endothelial cells. Fluorescence intensity of phosphorylated H2A.X immunostaining indicating DNA double strand breaks in mouse primary endothelial cells 10 min, 60 min, 4 h and 24 h after exposure to 0, 0.1, 2, 10 Gy irradiation. Values presented are means ± SD, n = 6 from 2 separate experiments. Statistical analysis: two-way ANOVA followed by Bonferroni post-test. Statistically significant difference p<0.001 (*) and p<0.001 (***) is indicated. (TIF) [file pone.0112397.s004.tif]
