# Supplementary material for: Low Dose Cranial Irradiation-Induced Cerebrovascular Damage Is Reversible in Mice
Source: PLoS One. 2014 Nov 13;9(11):e112397. doi: 10.1371/journal.pone.0112397 (PMC4231057; doi:10.1371/journal.pone.0112397)
Supplement: Text S1 — (DOCX) [file pone.0112397.s005.docx]

# Supporting Information:

# Materials and Methods

DNA Repair Kinetics

The kinetics of the repair of DNA double breaks was followed by histone H2A.X phosphorylation measured by immunostaining. Flow cytometric analysis was based on the method of Kataoka et al. (79). MBECs were were grown to confluence in 6-well plates and irradiated. Cells were trypsinized with Trypsin/EDTA (Gibco, Life Technologies, USA) and collected in 1 ml culture medium 10 min, 60 min, 4 h and 24 h postirradiation. Cells were fixed with 2 % paraformaldehyde for 10 min and immediately cooled on ice, centrifuged, washed three times with 0.5 % BSA-PBS, permeabilized with 90 % methanol in 0.5 % BSA-PBS, and kept at -20°C until further staining. Cells (1–2×10^5^) were washed three times with 0.5 % BSA-PBS, stained with FITC labeled anti-phospho-histone H2A.X (phospho-Ser139) antibody (Millipore, USA) for 1 h at 4 °C in the dark, and then washed three times with 0.5 % BSA-PBS. Stained samples were analyzed by FACSCalibur flow cytometer (BD Biosciences, USA). 10,000 cells were analyzed on the flow cytometer per sample/ data point. Analysis was performed using CellQuest^TM^ software, version 3.1 (BD Biosciences, USA).
